# The Coevolution of IDO1 and AhR in the Emergence of Regulatory T-Cells in Mammals

**DOI:** 10.3389/fimmu.2015.00058

**Published:** 2015-02-12

**Authors:** Ursula Grohmann, Paolo Puccetti

**Affiliations:** ^1^Department of Experimental Medicine, University of Perugia, Perugia, Italy; ^2^Department of Pathology, Albert Einstein College of Medicine, New York, NY, USA

**Keywords:** aryl hydrocarbon receptor, tryptophan metabolism, indoleamine 2,3-dioxygenase 1 and 2, tryptophan dioxygenase, immune regulation

The aryl hydrocarbon receptor (AhR) is a ligand-operated transcription factor originally recognized as the mediator of the toxicity of dioxins. AhR is presumed to have evolved from invertebrates, where it served a ligand-independent role in normal development processes. Evolution of the receptor in vertebrates resulted in the ability to bind structurally different ligands, including xenobiotics, such as dioxin, and catabolites derived from the host’s own metabolism, or from the microbiota. It is now clear that AhR contributes to immune homeostasis by promoting immunomodulatory and host-protective effects (1, 2). It is likewise clear that the nature of the ligand as well as the tissue specificity [e.g., gut (3), skin (4), and lymphoid tissue] in which AhR engagement occurs largely dictate the outcome of AhR activation.

Indoleamine 2,3-dioxygenase 1 (IDO1) catalyzes the initial rate-limiting step in degrading tryptophan along the kynurenine pathway (5–7). Initially confined to regulation of tryptophan availability in local tissue microenvironments, IDO1 is now considered to play a wider role that extends to homeostasis and plasticity of the immune system. Its effects involve not only tryptophan deprivation but also the production of immunoactive kynurenines, which may act as AhR ligands (8–12). Although two additional enzymes, i.e., IDO2 and tryptophan 2,3-dioxigenase (TDO2), catalyze the same reaction along the kynurenine pathway (13), IDO1 is apparently unique in promoting immunoregulatory effects over the long term, owing to its ability to function as a signaling molecule (14–16). IDO1 first appeared in placental animals by duplication of the *IDO2* gene (17), suggesting that the coexistence of two allogeneic individuals (i.e., mother and fetus) in the same organism would require advanced strategies of immune regulation capable of maintaining T-cell tolerance for prolonged periods of time.

The appearance of higher vertebrates, and specifically mammals, was, in fact, marked by the emergence of regulatory T (Treg) cells (18, 19). Thus, an entirely new paradigm in immunology, and more specifically in immune tolerance, may be the coevolution of three systems, namely, the IDO1 mechanism, kynurenine-driven gene transcription, and T-cell regulatory activity, which, originating from the initial need of protecting the fetus in mammals, have later turned into a pivotal mechanism of peripheral tolerance in autoimmunity, transplantation, and neoplasia.

The present Research Topic brings together 11 articles covering evolutionary aspects of tryptophan catabolic enzymes and AhR, their role in physiology and pathogenesis. In their Review Article, Ball et al. pointed out two interesting features emerging from studies of the dynamic evolution of TDO2, IDO1, and IDO2 (20). The three enzymes, which belong in two distinct superfamilies (TDO and IDO), have *converged* into the same catalytic activity, thus underlining the critical importance of tryptophan metabolism in all organisms. Yet, the IDO superfamily underwent *divergent* evolution, which occurred by gene duplication and led to the expression of an eclectic protein, IDO1, in placental animals. Because *Ido1*^−/−^ mice are mosaic deficient for the IDO2 function possibly owing to an altered mRNA splicing, distinct IDO genes may also influence the expression of each other by a still unclarified mechanism, as suggested by Prendergast et al. (21).

Zelante et al. dealt with the adaptive properties of IDO1 and AhR from a different perspective, i.e., taking into consideration the possibility that tryptophan metabolism by human microbiome has been playing a major role in shaping the coevolution of the AhR/IDO1 axis in immune regulation (22). Interestingly, they recently discovered a tryptophan catabolite selectively produced by certain *Lactobacilli* of the human microbiome (i.e., indole-3-aldehyde) capable of activating AhR and thus inducing the expression of IDO1 and anti-inflammatory responses (3). Tryptophan catabolic enzymes may, however, represent a double-edged sword in the interaction between mammals and pathogenic microbes, as outlined by Schulze et al. (23), because tryptophan depletion exerts bactericidal activity in tryptophan auxotrophs. Microorganisms such as *E. coli* and HIV are known to highjack the immunosuppressive effects of IDO1, though. Intriguingly, Kishimoto et al. discussed the possibility that microRNAs (miRNAs) may regulate the transcriptional expression of IDO-encoding genes, mainly in autoimmunity (24). Because miRNAs have been suggested to be instrumental in the evolution of organismal complexity (25) and AhR has been shown to induce the expression of several miRNAs (26), these observations further underline the critical interdependence of AhR and IDO enzymes in coping with mammalian challenges.

In their Perspective, Orabona et al. proposed an additional level of cross-regulation between the two systems (27), which may occur via AhR non-genomic effects that imply recruitment of a ubiquitin ligase complex and consequent proteasomal degradation of target proteins, a mechanism also considered by Quintana et al. (28). Because IDO1 is known to be subjected to regulatory proteolysis, AhR may not only induce but also switch off the IDO1 mechanism. Thus, depending on the specific pathologic conditions and timing of events, AhR may represent a friend or foe, and pollutants may play a major role in this regard, as suggested by the Perspective Article by Mezrich et al. (29).

Neoplasia represents a condition where drug targeting of the AhR/tryptophan metabolism axis has made the greatest progress. Already considered as a mechanism of immune escape in tumor progression, the data by Hanks et al. indicate that IDO1, modulated by several factors, is also involved in creating the permissive conditions for early carcinogenetic events (30). Most of these AhR/IDO1 modulating factors were examined by Platten et al. as potentially indirect, yet innovative, drug targets (31). Van den Eynde et al., thanks to the use of a highly specific anti-human IDO1 antibody, elegantly revisited the expression of this immunoregulatory enzyme in almost one thousand tumor specimens, finding that IDO1 is not upregulated in tumor-draining lymph nodes as previously reported, but it is restricted to tumor cells, stroma, and endothelium (32).

The reviews presented in this e-book of Frontiers are meant to provide readers with an overview of the intricacies of AhR functioning in both physiology and pathology, and of the combined effects of AhR ligand – intrinsic and – extrinsic factors, including the local tissue, which may provide a specific set of coactivators and functions bridging the basic transcriptional machinery to the target genes.

## Conflict of Interest Statement

The authors declare that the research was conducted in the absence of any commercial or financial relationships that could be construed as a potential conflict of interest.
